# Optimized riboswitch-regulated AAV vector for VEGF-B gene therapy

**DOI:** 10.3389/fmed.2022.1052318

**Published:** 2022-12-13

**Authors:** Reetta A. E. Eriksson, Tiina Nieminen, Lionel Galibert, Sanna K. Peltola, Petra Tikkanen, Piia Käyhty, Hanna M. Leinonen, Igor Oruetxebarria, Saana Lepola, Anniina J. Valkama, Eevi M. Lipponen, Hanna P. Lesch, Seppo Ylä-Herttuala, Kari J. Airenne

**Affiliations:** ^1^Kuopio Center for Gene and Cell Therapy, Kuopio, Finland; ^2^A.I. Virtanen Institute for Molecular Sciences, University of Eastern Finland, Kuopio, Finland; ^3^Gene Therapy Unit and Research Center, Kuopio University Hospital, Kuopio, Finland

**Keywords:** riboswitch, ON-switch, gene therapy, AAV (adeno-associated virus), VEGF-B, tetracycline, transgene expression regulation

## Abstract

Gene therapy would greatly benefit from a method to regulate therapeutic gene expression temporally. Riboswitches are small RNA elements that have been studied for their potential use in turning transgene expression on or off by ligand binding. We compared several tetracycline and toyocamycin-inducible ON-riboswitches for a drug responsive transgene expression. The tetracycline-dependent K19 riboswitch showed the best control and we successfully applied it to different transgenes. The induction of gene expression was 6- to 10-fold, dose-dependent, reversible, and occurred within hours after the addition of a clinically relevant tetracycline dose, using either plasmid or adeno-associated virus (AAV) vectors. To enhance the switching capacity, we further optimized the gene cassette to control the expression of a potential therapeutic gene for cardiovascular diseases, *VEGF-B*. Using two or three riboswitches simultaneously reduced leakiness and improved the dynamic range, and a linker sequence between the riboswitches improved their functionality. The riboswitch function was promoter-independent, but a post-transcriptional WPRE element in the expression cassette reduced its functionality. The optimized construct was a dual riboswitch at the 3′ end of the transgene with a 100 bp linker sequence. Our study reveals significant differences in the function of riboswitches and provides important aspects on optimizing expression cassette designs. The findings will benefit further research and development of riboswitches.

## Introduction

Although gene therapy has taken significant leaps forward in the past decades, it still largely lacks a method to regulate transgene expression temporally. With an uncontrolled expression, for example growth factor-based therapies can induce toxicity, which might prevent potential treatments (1, 2). The ability to regulate transgene expression would benefit gene therapies and increase potential targets. However, developing efficient regulation mechanisms has proven challenging; to date, none have reached clinical trials.

One interesting possibility of controlling transgene expression in gene therapy is utilizing riboswitches, small RNA elements regulating gene expression post-transcriptionally. Natural riboswitches were first identified to control genes in bacteria by repressing expression upon ligand binding (3), but soon also in eukaryotes (4), and inducing gene expression (5). Synthetic aptazyme riboswitches consist of two domains: an aptamer for the ligand binding, and a ribozyme. This composition is of a modular nature; domains can be changed while maintaining the riboswitch function (6).

**FIGURE 1:**
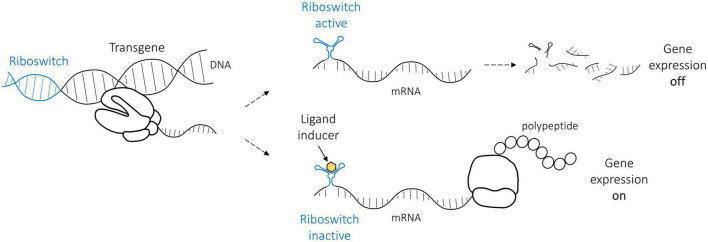
The basic principle of the ON-riboswitch regulation used in this study. In its native RNA form, the riboswitch (indicated in blue) self-cleaves and thus leads to the cleavage of the messenger RNA (gene expression is off). Riboswitch structure is altered, however, when a specific ligand (yellow) such as tetracycline binds it, and the gene expression may proceed (gene expression on).

Riboswitches regulate gene expression by inducing (ON-switches) or repressing (OFF-switches) gene expression. For practical reasons, we focused on ON-riboswitches that, in their native form, cause the degradation of the mRNA, thus stopping gene expression (Figure 1). The riboswitch self-cleaves, which removes the poly(A) tail from the rest of the mRNA, finally resulting in mRNA degradation. However, ligand binding alters riboswitch folding, preventing the cleavage, which enables gene expression to continue. In a clinical application, a patient will receive gene transfer, and only the administration of the ligand induces and maintains transgene expression for a desired treatment time. Not all gene therapy benefits from this kind of a regulation; however, in many cases, a riboswitch could increase safety by enabling switching off the transgene expression.

Besides riboswitches, also other transgene regulation systems have been proposed, such as RNA-based regulation by alternative splicing (7), destabilizing domains for control by protein degradation (8), and the well-known Tet On/Off system (9). Riboswitches, however, have several advantages that make them an attractive option for gene therapy. Firstly, their small size of about 100 bp enables them to fit into even small viral vectors like adeno-associated virus (AAV) vectors. Secondly, the riboswitch function is highly ligand-specific; our tetracycline-binding riboswitch shows no response to the very similar doxycycline (10). Thus, unspecific binding and accidental switching on or off is unlikely. Finally, unlike some other common switching methods, like Tet On/Off, riboswitches require no protein co-expression. Riboswitch regulation is direct and rapid, as no intermediate factors need to be produced. Furthermore, the need for these recombinant transcription factors, often of foreign origin, raises safety concerns (11). Especially when translating these systems to non-human primates, the immune reactions may interfere with the therapy (12). Indeed, riboswitches and other RNA elements are a rather feasible and safe option as they fit well to any vector and the risk of detection by the immune system is lower.

Riboswitches were first utilized to control transgene expression in mammalian cells by Yen et al. (13). Since then, different groups have designed riboswitches responding to various ligands. Both ON and OFF-switches have been developed. While OFF-switching is simpler and has in general worked better in the transgene regulation context than ON-switches (14), most applications would benefit from the ability to switch on the expression. Few publications have shown riboswitch-regulated transgene expression *in vivo* (13–16). These studies have reported riboswitch regulation in different mouse tissues, such as the eye (13, 15), skeletal muscle (14, 16), liver, and heart (16).

We aimed to develop a treatment for ischemic heart utilizing the vascular endothelial growth factor B (VEGF-B). It is a VEGF family member that increases the metabolism of heart muscle and induces vascularization (17), and could thus be beneficial in heart failure treatments (18). However, the issue with VEGF-B and other growth factor-based gene therapies is currently that if expressed constantly, they can lead to adverse effects. Therefore, developing a regulation method is crucial for these therapies.

To find the best clinically compatible ON-switch for regulated VEGF-B gene therapy, we compared several riboswitches (10, 13, 19–21), for many of which no previous comparisons have been reported. We also used different transgenes to maximize the comparability and applicability of the results. The best design showed a tetracycline-induced transgene expression of up to 10-fold. The induction was dose-dependent and reversible, and started within hours after introducing tetracycline to cell culture. We used this riboswitch to control the expression of three different transgenes: destabilized enhanced green fluorescent protein (*d2EGFP*), murine secreted alkaline phosphatase (*muSEAP*), and *VEGF-B186*.

To further understand and optimize the system, we also modified the *VEGF-B* gene cassette components. The regulation functioned despite most changes made to the cassette. Some modifications increased the dynamic range while others decreased it. Finally, we inserted the regulated *muSEAP* and *VEGF-B186* cassettes into recombinant AAV vectors and showed that they sustain the switching capability. Our results demonstrate that riboswitches are reliable and flexible regulators of gene expression.

## Results

### Riboswitch constructs respond to inducer variably

We tested 11 different riboswitch-controlled expression cassettes encoding the destabilized *EGFP* as a marker gene. Plasmids for different riboswitch comparison experiments contained cytomegalovirus (CMV) enhancer, CMV promoter, a chimeric adenovirus intron, and the *d2EGFP* transgene. We compared seven different riboswitches, some at both 5′ and 3′ ends of the transgene. Supplementary Table 1 lists all the plasmids with the details of the riboswitches.

**FIGURE 2:**
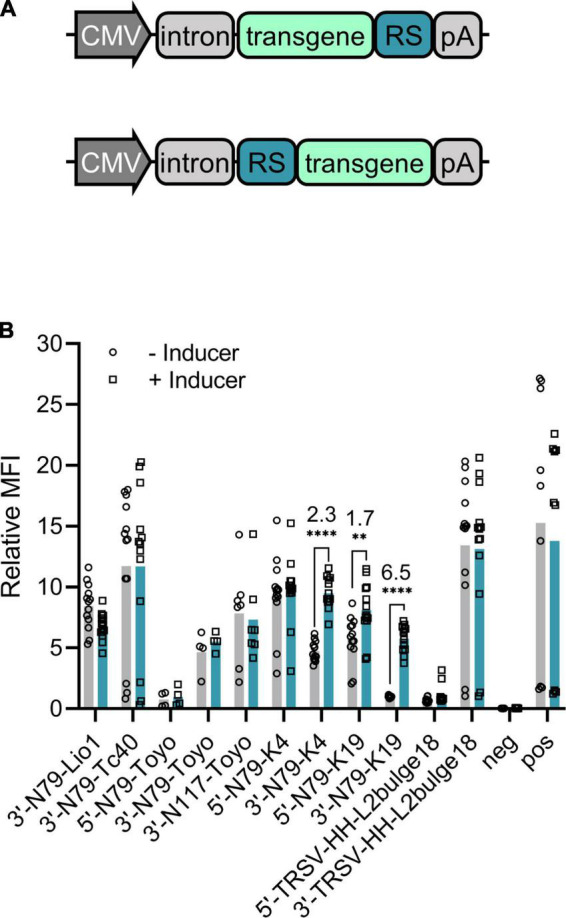
Riboswitch construct comparison. **(A)** General schematic maps of 3′ (top) and 5′ (bottom) riboswitch (RS) constructs also used in the following experiments. **(B)** Destabilized EGFP regulated by different riboswitch constructs in plasmid-transfected 293T cells. Group mean values are indicated with bars and individual data points with symbols, all as fold-changes to 3′N79-K19 with no inducer. For the constructs with a statistically significant induction (Šídák’s multiple comparisons test), the CDR (corrected dynamic range) is given in the figure. Pos refers to a d2EGFP-coding plasmid with no riboswitch sequence, and neg to a d2EGFP plasmid with no active promoter. *N* = 13 (five separate experiments), except for N79-Toyo constructs, where *N* = 4 (two separate experiments), and 3′-N117-Toyo, where *N* = 7 (three separate experiments). **p < 0.01, ****p < 0.0001. MFI, mean fluorescence intensity.

Most of the constructs showed little or no response to the inducer (Figure 2B). However, we identified one, 3′-N79-K19, that functioned well showing a 6.5-fold corrected dynamic range (CDR; calculated as described in Zhong et al. (19) by dividing the ligand-induced fold-change by the fold-change in the positive control). The same riboswitch at the 5′ end of the transgene only resulted in a CDR of 1.7, with a rather high leakage (expression level with no inducer) when compared to that of the 3′ construct. Likewise, the second-best riboswitch construct N79-K4 reached a CDR of 2.3 when located at the 3′ end, but the 5′ construct had higher expression levels and no significant response to the inducer. While most of the other constructs had lower expression than the positive control plasmid with no riboswitch, the inducers had no effect on their transgene expression.

### Riboswitch function is transgene-independent

**FIGURE 3:**
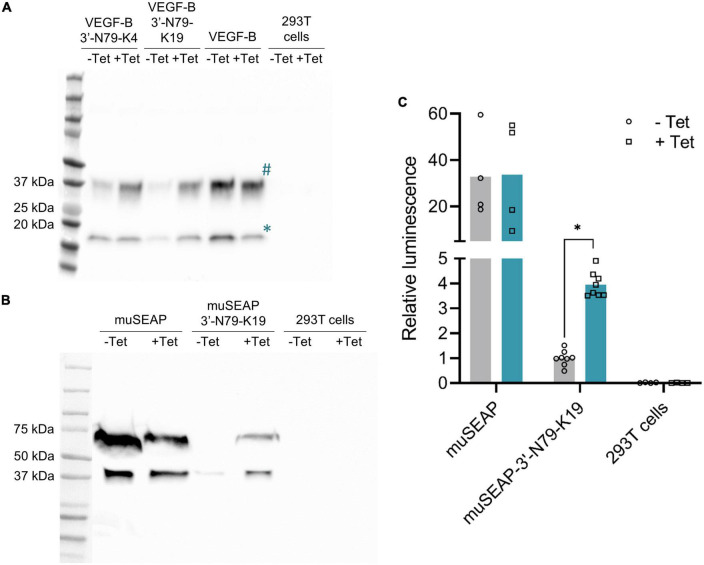
Riboswitch regulation is transgene-independent. **(A)** Western blot showing VEGF-B186 expression controlled with the 3′-N79-K4 and K19 riboswitches. VEGF-B186 protein is seen as the band at 37 kDa (annotated #), and the proteolytically processed 127 form below 20 kDa (annotated *). **(B)** Western blot showing murine secreted alkaline phosphatase (muSEAP) expression. **(C)** MuSEAP quantified from plasmid-transfected cell media with the chemiluminescent assay. Analyzed with Šídák’s multiple comparisons test. *N* = 4 for controls (two separate experiments), *N* = 6 for the riboswitch constructs (three separate experiments). *p < 0.05.

We applied the two most promising riboswitches, 3′-N79-K4 and 3′-N79-K19, to control the expression of *VEGF-B186* (Figure 3A). Tetracycline increased gene expression from both plasmids. The results were similar to those of *d2EGFP*, 3′-N79-K19 showing a better dynamic range and lower leakiness. Finally, we cloned the 3′-N79-K19 riboswitch element to control the expression of *muSEAP* (Figures 3B,C), proving that the regulation is independent of the transgene. In the positive control plasmids, tetracycline often seemed to decrease protein levels, as seen in Figures 3A,B.

### Riboswitch regulation is dose-dependent

**FIGURE 4:**
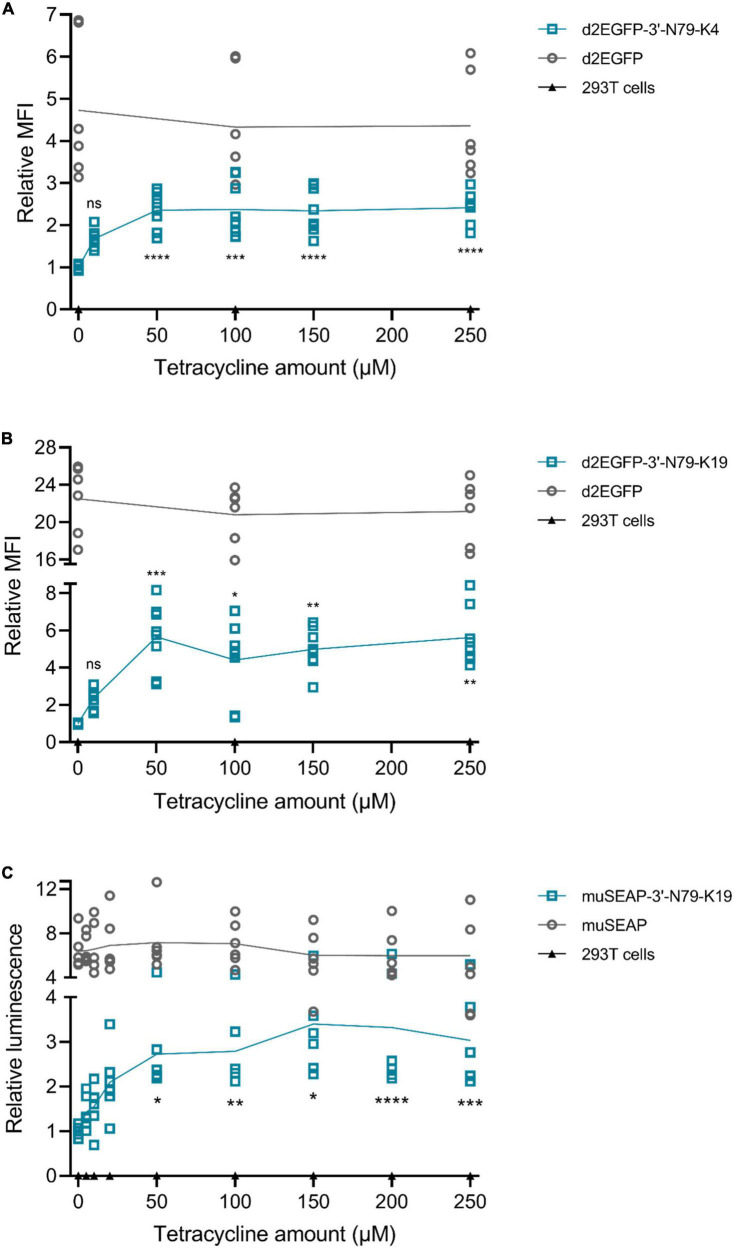
Tetracycline dose dependency in plasmid-transfected 293T cells. Mean fold-changes in mean fluorescence intensity to 0 μM tetracycline in the riboswitch construct and individual data points indicated, as well as the statistical significance to 0 μM. **(A)** d2EGFP-3′-N79-K4 and controls. *N* = 6 (three separate experiments). **(B)** d2EGFP-3′-N79-K19 and controls. *N* = 8 (four separate experiments). Tet concentrations for *d2EGFP* plasmids: 0, 10, 50, 100, 150, and 250 μM. **(C)** muSEAP-3′-N79-K19 and controls. Tet concentrations for murine secreted alkaline phosphatase (muSEAP) plasmids: 0, 5, 10, 20, 50, 100, 150, 200, and 250 μM. Concentrations lower than 50 had no statistically significant difference to 0 μM. *N* = 6 (three separate experiments). Tukey’s multiple comparisons test. ns, non-significant, *p < 0.05, **p < 0.01, ***p < 0.001, ****p < 0.0001.

By adding different concentrations of tetracycline into cell culture, we found that the protein expression is indeed dependent on the dose of tetracycline (Figure 4). For both 3′N79-K4 and K19 riboswitches controlling *d2EGFP* expression, as well as K19 with *muSEAP*, a slight increase was visible already at low tetracycline concentrations of 5 and 10 μM, although statistical significance was found at 50 μM. The protein levels plateaued after 50 μM.

### Riboswitch regulation is rapid and reversible

**FIGURE 5:**
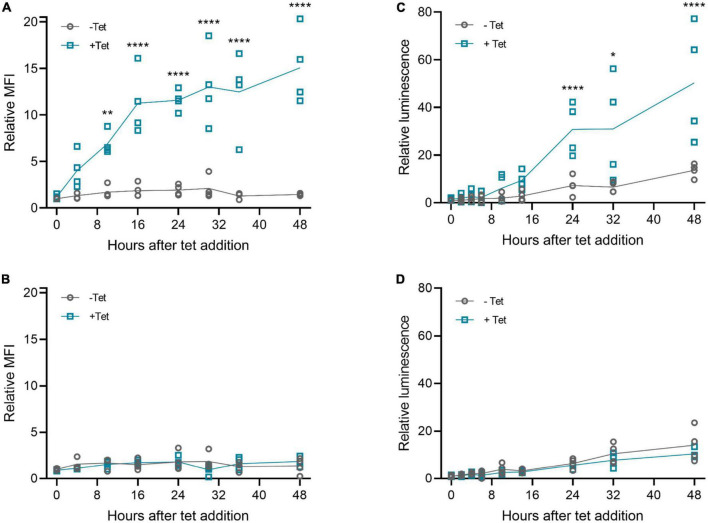
Riboswitch induction kinetics with plasmid transfections in 293T cells. Tetracycline (100 μM) was added to cells 20 h post-transfection, and cells (d2EGFP) or media (muSEAP) were collected at different time points thereafter. **(A)** d2EGFP-3′-N79-K19 and **(B)** d2EGFP positive control plasmid. Relative fluorescence intensity to 0 h samples without tetracycline indicated. *N* = 4 (two separate experiments). **(C)** muSEAP-3′N79-K19 and **(D)** muSEAP positive control plasmid. Relative luminescence to 0 h samples without tet indicated. *N* = 4 (two separate experiments). Šídák’s multiple comparisons test. Statistically significant differences between –tet and +tet are shown (non-significant differences not indicated). *p < 0.05, **p < 0.01, ****p < 0.0001.

Next, we examined the kinetics of tetracycline-induced protein expression with the 3′-N79-K19 riboswitch. As Figure 5 shows, tetracycline started to induce the expression within a few hours after adding it. The induction was slightly slower with muSEAP (Figure 5C) than d2EGFP (Figure 5A). The earliest time point with a statistically significant difference between the cells with and without tetracycline was at 10 h after addition with the *d2EGFP* plasmid, and at 24 h with *muSEAP*. The expression level increased linearly until about 16 h with *d2EGFP*, and 24 h with *muSEAP*.

**FIGURE 6:**
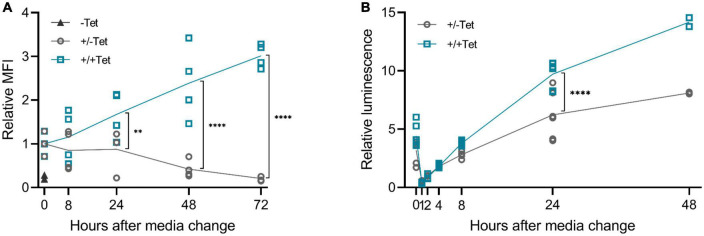
The effect of tetracycline retraction in 293T cells with the 3′-N79-K19 riboswitch. Tetracycline (100 μM) was added to the cells as usual, but after 24 h, cell culture media was changed. The cells were also washed with PBS. Half of the cells received fresh media with tetracycline, at the same concentration as before, and the rest received media with no tetracycline. A statistically significant difference between the expression of the cells with the added tetracycline (+/+Tet) and those without (±Tet) was found at 24 h after media change both in d2EGFP and murine secreted alkaline phosphatase (muSEAP) with Šídák’s multiple comparisons test. **(A)** d2EGFP. By 72 h, the mean fluorescence intensity (MFI) of the cells with tet removed (±Tet) matched that of the cells entirely without tet (–Tet). *N* = 4 (two separate experiments). **(B)** muSEAP. *N* = 6 (three separate experiments), except for 48 h time point, *N* = 2 (one experiment). **p < 0.01, ****p < 0.0001.

We then wanted to study the reversibility of the tetracycline induction, as it is a crucial aspect for potential applications in gene therapy. Our results showed that after removing the inducer, the expression diminishes (Figure 6). Differences between d2EGFP and muSEAP indicate the different half-lives of the two proteins. While muSEAP accumulates over time (22) (Figure 6B), d2EGFP mimics the real-time protein expression level (23) (Figure 6A). Nevertheless, both showed a statistically significant difference in protein levels between the groups with and without tetracycline at 24 h after media change. The decrease of muSEAP in early time points, as visible in Figure 6B, is due to the media change, as muSEAP is a secreted protein. Most likely, some tetracycline remains inside the cells, which explains the production of both muSEAP and d2EGFP after removing tetracycline. The difference in the amounts of the two proteins after removing tetracycline is due to the long half-life of muSEAP.

### Woodchuck hepatitis virus post-transcriptional regulatory element effect depends on positioning

**FIGURE 7:**
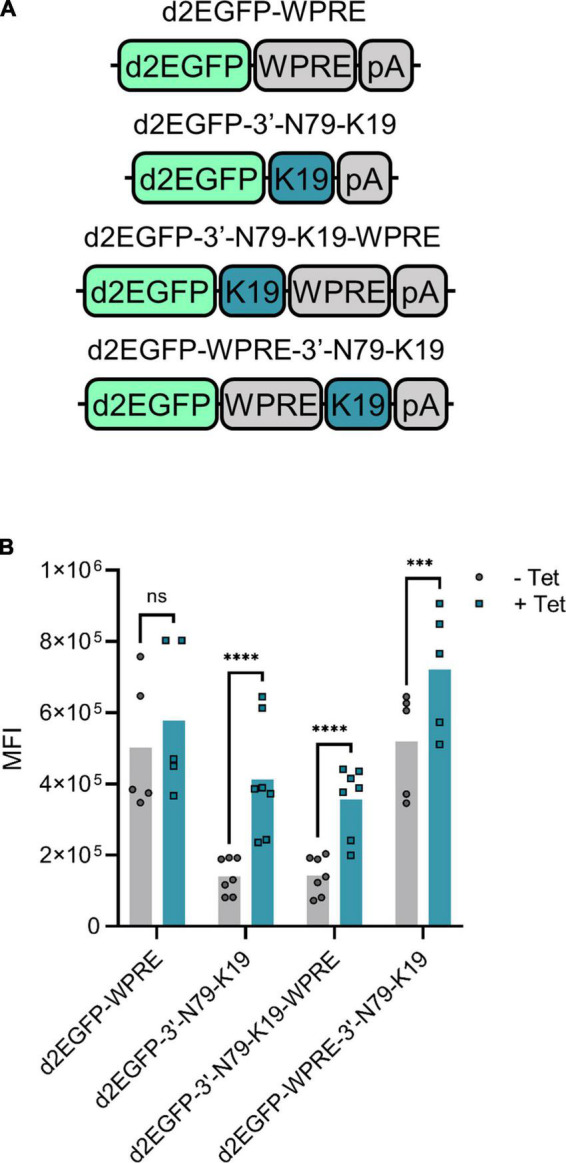
The effect of Woodchuck hepatitis virus post-transcriptional regulatory element (WPRE) on riboswitch regulation. **(A)** The compared *d2EGFP* constructs. K19 refers to N79-K19. **(B)** Mean fluorescence intensity (MFI) levels of the transfected 293T cells with and without tetracycline. For d2EGFP-WPRE and WPRE-3′-N79-K19, *N* = 5 (two experiments), and for 3′-N79-K19 and 3′-N79-K19-WPRE, *N* = 7 (three experiments). Šídák’s multiple comparisons test. ns, non-significant, ***p < 0.001, ****p < 0.0001.

The Woodchuck Hepatitis Virus Post-transcriptional Regulatory Element (WPRE) enhances mRNA stability and thus, increases protein amounts (24). We added a WPRE to our *d2EGFP* expression cassette, either between the transgene and the riboswitch, or after the riboswitch (Figure 7A). The overall expression increased with the WPRE between the transgene and the riboswitch (Figure 7B), but simultaneously, the induction between the cells without and with tetracycline greatly decreased. When added after the riboswitch, however, the WPRE had no effect on the d2EGFP amounts.

### Codon optimization of VEGF-B186 increases protein production

To increase protein expression levels, we tested the effect of codon optimization on *VEGF-B186* with no riboswitch. For a further increase, a WPRE was added to the expression cassette. We compared these plasmids with the codon-optimized *VEGF-B186* to the original one. VEGF-B186 protein production was increased by codon optimization, and further by the WPRE (Supplementary Figure 1A). Based on BaF3-R1 cell growth and survival assay, all these VEGF-B186 constructs were also biologically active (Supplementary Figure 1B). Mock-transfected cell medium also increased the viability of the BaF3-R1 cells at high concentrations, probably due to the endogenous production of VEGFs in 293T cells (25, 26).

### Leakiness increases with expression rate

**FIGURE 8:**
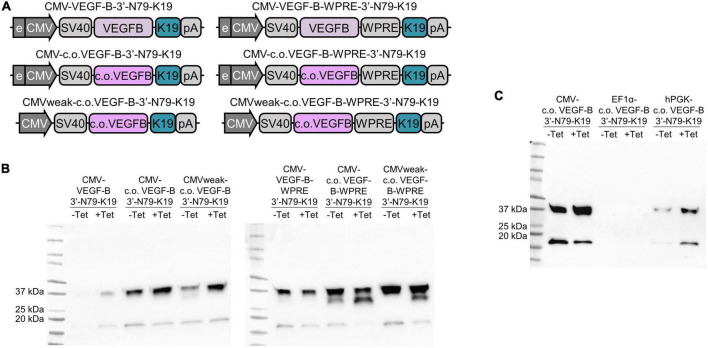
Promoter modification or change, Woodchuck hepatitis virus post-transcriptional regulatory element (WPRE), and codon optimization combined effects on riboswitch function. **(A)** CMV weak refers to the CMV enhancer/promoter from which the enhancer is mostly removed. **(B)** CMV promoter-driven expression of normal and codon-optimized (c.o.) *VEGF-B186* with and without the CMV enhancer and WPRE element **(C)** CMV, EF1α, and hPGK promoters with codon-optimized *VEGF-B186*.

Next, we focused on the modification of the riboswitch-controlled *VEGF-B186* construct to increase its expression levels. Both the VEGF-B186 codon-optimization and the WPRE greatly increased VEGF-B186 amounts (Figure 8B, left and right blot). The protein expression was the highest when these two modifications were combined (Figure 8B, right blot). However, the leakiness of the constructs was also increased, so we next focused on reducing the leakiness by several means.

Aiming to reduce leakiness of the modified riboswitch constructs with high expression levels, we removed most of the CMV enhancer from the original promoter (Figure 8A). We speculated that this could slow down transcription and enable the riboswitch to fold more efficiently. This weakened CMV promoter somewhat decreased the leakiness of the codon-optimized 3′-N79-K19-VEGF-B186 (Figure 8B, left blot), but on the WPRE-containing construct, it had no distinctive effect (Figure 8B, right blot).

We also changed the CMV promoter driving the codon-optimized *VEGF-B186* expression to other weaker ones, human elongation factor-1 alpha (EF1α) and human phosphoglycerate kinase (hPGK). The hPGK lowered leakiness, but also the overall expression level (Figure 8C). From the EF1α construct, VEGF-B186 was initially not detected; however, with larger doses of tetracycline, it was visible on Western blot (Supplementary Figure 2).

### Multi-riboswitch constructs maintain the system functionality

**FIGURE 9:**
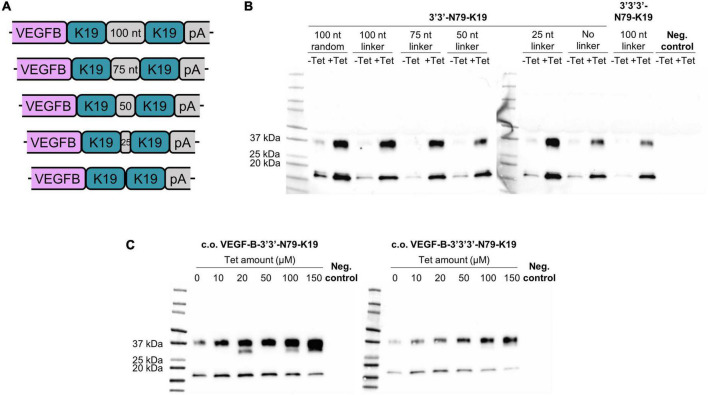
Double and triple riboswitch-controlled *VEGF-B186* expression in 293T cells. **(A,B)** Linker comparison with 3′3′-N79-K19. **(C)** Double (left blot) and triple (right blot) 3′-N79-K19-riboswitch constructs controlling the codon-optimized *VEGF-B186*, induced by different doses of tetracycline.

To further reduce leakiness while maximizing the protein expression, we increased the copy-number of 3′-N79-K19 riboswitches to two or three subsequent elements. Additionally, in the dual-riboswitch construct, we studied the length requirements and sequence specificity of the linker sequence between the riboswitches. The tested linker sequences were 0, 25, 50, 75, 100, and a random 100 bp sequence that had no sequence homology to the other linkers (Figure 9A). Linker sequences are given in Supplementary Table 2. The triple-riboswitch construct had the non-random 100 bp linker. Linker length played no significant role in the switching; however, a linker was favorable, as the construct with no linker had a slightly reduced induction (Figure 9B). In the plasmids with the codon-optimized *VEGF-B186*, the dual and triple 3′-N79-K19 riboswitch constructs with the 100-nucleotide linker maintained the dose-dependent nature (Figure 9C).

### Dual and triple riboswitch elements reduce the leakiness and increase the dynamic range of the codon optimized VEGF-B186

**FIGURE 10:**
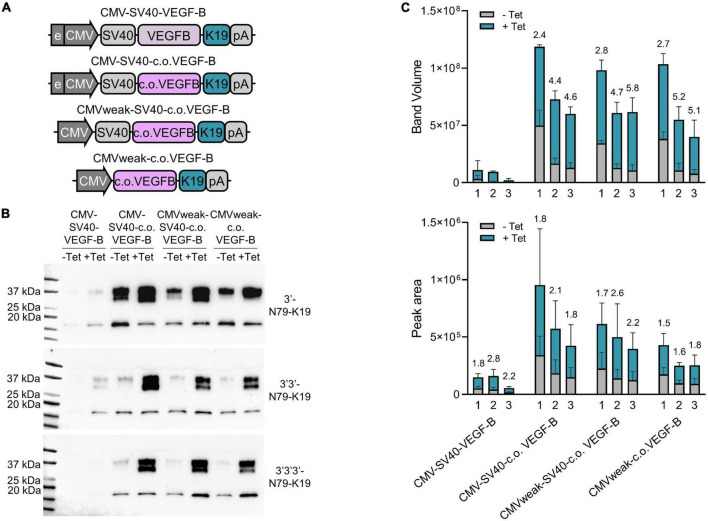
Single, double, and triple riboswitch constructs with different modifications. **(A)** Changes made to the plasmids. First, in the original plasmid, the *VEGF-B186* was changed to the codon-optimized version. Next, the CMV enhancer was removed, and, finally, the SV40 intron was removed. All these changes were made in single, double, and triple 3′-N79-K19 plasmids. **(B)** Western blots showing the protein amounts produced from different plasmid-transfected 293T cells. **(C)** Band volumes from Western blots [top; *N* = 3 (three separate experiments)] and peak areas from Jess simple western [bottom; *N* = 6 (three separate experiments)]. Single (1), double (2), and triple (3) riboswitch version of each plasmid backbone variation. Mean band volumes or peak areas and standard deviations are indicated, as well as, above the bars, fold-changes between –Tet and +Tet.

Finally, we systemically compared the single, dual, and triple 3′-N79-K19 constructs regulating the expression of *VEGF-B186* (Figure 10). Constructs were compared to the original plasmids that we modified. First, *VEGF-B186* was changed to the codon-optimized version. Next, we removed the CMV enhancer, and, finally, the SV40 intron. In single, dual, and triple riboswitch constructs, VEGF-B186 amounts greatly increased with codon-optimization. Leakiness, along with the overall expression, was reduced with the two and three riboswitch constructs. Here, removing the CMV enhancer and SV40 intron further reduced the leakiness. Importantly, while two or three riboswitches lowered the overall VEGF-B186 expression levels, compared to the single riboswitch constructs, the dynamic ranges were better.

### Riboswitch function and characteristics are sustained in inverted terminal repeat plasmids and rAAV6 vectors

**FIGURE 11:**
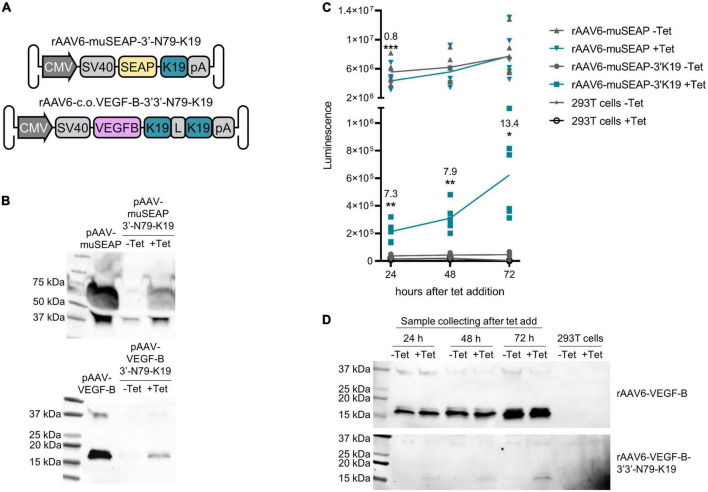
Riboswitches controlling ITR plasmid and viral vector-mediated gene expression in 293T cells. **(A)** Riboswitch-regulated murine secreted alkaline phosphatase (muSEAP) (top) and codon-optimized VEGF-B186 (bottom) vector genomes. *L*, linker. **(B)** muSEAP (top) and VEGF-B186 (bottom) protein expression quantified by Western blot from ITR plasmid-transfected cell media. **(C)** muSEAP quantified from rAAV6 vector-transduced cell media. Transductions with vectors with and without the riboswitch. Cells were collected 24, 48, and 72 h after adding tetracycline. The graph shows the mean and individual luminescence values. Corrected dynamic ranges (CDRs) of the riboswitch vector at each time point are indicated in the figure, as well as the fold-change (–tet to +tet) of a statistically significant difference found in the control vector at 24 h. Tukey’s multiple comparisons test. *N* = 6 (two experiments). **(D)** VEGF-B186 quantified with Western blot from rAAV6-transduced cell media. Same experimental setting as with the muSEAP vector. Upper blot shows the positive control vector with no riboswitch, and lower the double 3′-N79-K19 riboswitch-controlled vector. The two blots were imaged with different exposure times: 53 s for the positive control and 480 s for the riboswitch vector blot. *p < 0.05, **p < 0.01, ***p < 0.001.

**FIGURE 12:**
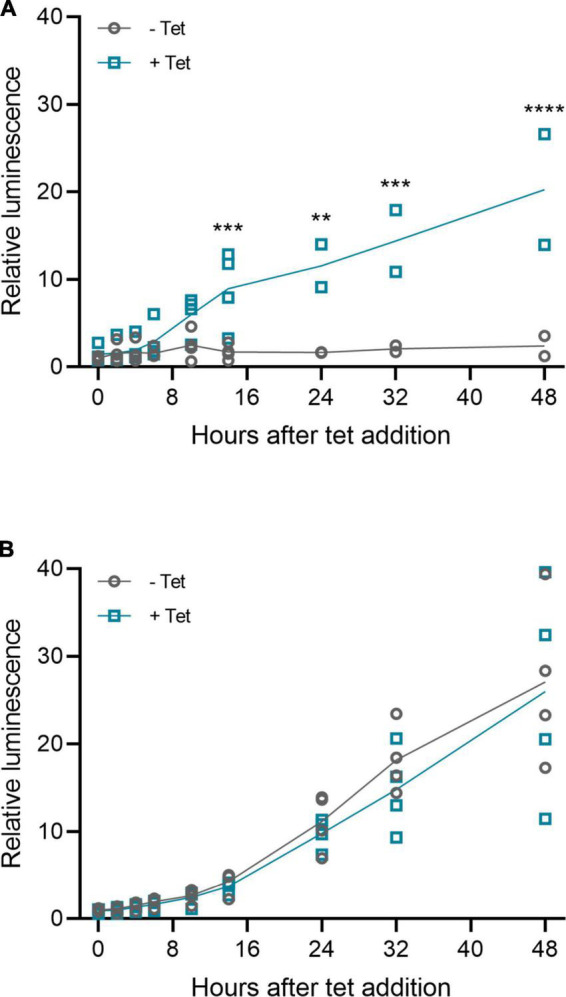
Murine secreted alkaline phosphatase (muSEAP) expression kinetics after adding tet inducer to rAAV6-transduced 293T cells. Media samples collected at 0, 2, 4, 6, 10, 14, 24, 32, and 48 h after adding tetracycline. **(A)** rAAV6-muSEAP-3′N79-K19 and **(B)** rAAV6- muSEAP. *N* = 4 (two separate experiments), except for rAAV6-muSEAP-3′N79-K19, where at time points starting at 24 h, *N* = 2 (one experiment). Values relative to the –tet 48 h luminescence. Šídák’s multiple comparisons test. Statistically significant differences between –tet and +tet at each time point shown. **p < 0.01, ***p < 0.001, ****p < 0.0001.

The riboswitches functioned also when cloned to inverted terminal repeat (ITR) plasmids and rAAV6 vectors, as shown in Figure 11 with the *muSEAP* and *VEGF-B* transgenes. The vector analyses (titering and Western blot) are described in Supplementary Figure 3. The induction of expression with the *muSEAP*-coding rAAV6 after adding tetracycline was similar to that of the corresponding plasmid (Figure 12; compare Figure 12A to Figure 5C).

## Discussion

In this work, we attempted to find an ON-switch for angiogenesis gene therapy. We compared the best riboswitches described in literature for their performance to control the transgene expression and evaluated ways to apply them to reduce leaky transgene expression while maximizing the dynamic range.

While some of our results were in line with previous research, most constructs showed no response to the ligand inducer. Riboswitch function can be sensitive to different factors, such as transduction/infection conditions like the *in vitro* magnesium ion concentration, or even single-nucleotide changes (13). Thus, some differences between our results and those of others were expected. The toyocamycin aptamer with the N79 and N117 ribozymes previously showed induction already at 1.5 μM toyocamycin (13); however, we saw no effect with 5 μM. Our Tc40 with the N79 ribozyme had a high overall expression and showed no response to tetracycline. However, it was originally published in the combination with a different ribozyme than the one we used (19). L2Bulge18tc was originally published in Chen et al. (20), where it showed a dose-dependent induction of *EGFP*; in another report, it showed induction in HEK293T cells (15). Our 3′ version, however, had an expression level very close to that of the positive control plasmid with no riboswitch. Evidently, this 3′ construct, in our hands, failed already at the mRNA cleavage. Interestingly, many of our compared riboswitches still lowered d2EGFP protein expression (compared to the positive control plasmid with no riboswitch); only the response to the ligand inducer was missing.

The 3′ K4 and 3′ K19 aptamers with the N79 ribozyme had similar fold-changes (CDR of 2.3 with K4, and 6.5 with K19) as in the original publication, as well as a similar expression (K19 lower than K4) when compared side-by-side (10). Our results showed the N79-K19 to function in 5′, though with a low induction rate. This conflicts with (16), where no response to tetracycline in this position was seen. Our results of tetracycline induction dynamics are in line with the previous published results (16), and complemented these, showing the protein expression for long time points.

We found slightly varying results between the transgenes. The difference between muSEAP and d2EGFP in the accumulation rate is likely explained by their different half-life. The destabilized EGFP with a short half-life of approximately 2 h (23) better shows the real-time expression level, while muSEAP accumulates with its *in vitro* half-life of 10–11 days (22). This resulted in different kinetics after removing tetracycline. A difference was also seen in the kinetics of the tetracycline induction between these proteins: muSEAP expression, both plasmid and rAAV-driven, began slower than d2EGFP. For further *in vivo* work, however, *muSEAP* is a good marker gene, as the protein is secreted into blood and thus, simple to monitor during a study.

To our knowledge, no previous studies have been published on the compatibility of the WPRE with riboswitches. For the function of the WPRE, the position in the gene cassette can be crucial (27), as was also demonstrated in this study. When the WPRE was inserted between the 3′ riboswitch and poly(A) sequence, the WPRE no longer increased the transgene expression. Here, the riboswitch seemed to interfere with the WPRE function. On the other hand, the WPRE between the transgene and the 3′ riboswitch increased expression, but the dynamic range of the tetracycline induction was lower. Thus, the WPRE seems to interfere with the riboswitch function, possibly affecting proper folding of the mRNA essential for riboswitch function.

Previous studies have shown varying results from using multiple riboswitches. Some have found an increased induction rate (28), while others found reduced overall expression levels with no benefit for the dynamic range (10, 16). One study found the preferable copy-number of riboswitches to be dependent on the used switch (15): the K19 riboswitch had the best induction with one copy; increasing the copy-number to up to four decreased the fold-change. Our results, however, showed an improvement in fold-change with two or three N79-K19 riboswitches. Some studies have inserted the riboswitches in both 3′ and 5′, which could improve the dynamic range (29); however, also merely overall reduced levels with no increase in fold-change, when compared to a single riboswitch, have been reported (16).

Although the best site for riboswitch embedding and using multiple riboswitch copies have been studied previously, to our knowledge, the effect of linker length between the multiple riboswitches has not been reported. While we found no major differences between the compared linkers, with no linker, the tetracycline-induced gene expression was slightly lower. Therefore, some linker between riboswitches is most likely preferable. We chose the 100 bp linker, but in some AAV vectors, a shorter linker could be applied to leave more space for other elements in the cassette.

Currently, the most used and clinically suitable riboswitch inducer is tetracycline. *In vivo*, it can be administrated in different ways using oral gavage and diet (15), intraperitoneal injection (16), drug-releasing pellets (13), or drinking water (30, 31). In a pharmacokinetic study in mouse following intraperitoneal administration, tetracycline was found in highest concentrations in liver, and less in the plasma, kidney, heart, skeletal muscle, and lung (16). However, in rodents, the bioavailability of tetracycline is poor, significantly lower than in humans, and especially low in mice (32). No riboswitch, to our knowledge, has been developed to respond to doxycycline. This tetracycline variant is pharmacologically better and has previously been successfully used in many Tet On/Off-based pre-clinical studies.

Only few studies have reported successful use of riboswitches *in vivo*, as their application in a living animal poses several challenges. These include the availability of only few inducers, as well as finding the best administration route and dosing frequency. In line, we had modest results in our previous studies using the non-optimized vectors. The successful translation of riboswitches to pre-clinical studies requires optimization and well-designed setups.

In conclusion, our side-by-side comparison of the best riboswitches described in the literature is partly in line with previous results. Indeed, riboswitches can control transgene expression in the context of different promoters, transgenes, and vectors, and the expression is dose-dependent and reversible. Surprisingly, however, only one riboswitch in well-optimized expression cassette gave minimized leakiness and good temporal dynamic range in controlling transgene expression. Riboswitch-regulated transgene expression is a promising strategy for a safe, dose-controlled gene therapy.

## Materials and methods

### Plasmids

All plasmids were produced by Genewiz (South Plainfield, New Jersey). In the riboswitches, the following ribozymes and aptamers were used: N79 and N117 Schistosoma mansoni hammerheads (SmHH) and toyo aptamer (13); tobacco ringspot virus (TRSV) hammerhead (21); Tc40 aptamer (19); K4 and K19 aptamers (10); and L2bulge18tc aptamer (20). One aptamer (Lio1) was our own design. For *muSEAP* and *VEGF-B186* plasmids, the gene cassette was slightly modified. This version was also used with *d2EGFP* in the tetracycline retraction experiments. Figure 2A presents a general schematic representation of these plasmids. Detailed information on all the plasmids is given in Supplementary Table 1.

Codon-optimization of *VEGF-B186* was done with the GeneArt algorithm (Thermo Fisher Scientific, Waltham, MA, USA). Integrated DNA Technologies (IDT), Coralville, Iowa produced the codon-optimized gene as a gBlock fragment that was cloned into existing backbones.

### Cell culture

293T cells (ECACC 12022001, RRID:CVCL_0063) were grown on multi-well plates in high-glucose Dulbecco’s Modified Eagle Medium (DMEM, Gibco 11965084) with 10% fetal bovine serum (FBS, Thermo Fisher 10091-148) and 1% Penicillin-Streptomycin (Gibco 15070-063). Cells were seeded a day prior to transfection or transduction. For transfections, 286 ng of plasmid was used per square centimeter of well area. PEIpro (Polyplus Transfection, Illkirch, France, 115-100) was mixed in DMEM with plasmid at 1 μg of PEIpro for 1 μg of plasmid DNA, and then incubated at room temperature for 15 min before adding to the cells. In transduction experiments, the vectors were used at 20,000 MOI (multiplicity of infection), diluted to an appropriate volume with DMEM. To enhance the transduction, an hour prior to it, Compound C (7.5 μM, Sigma-Aldrich, Saint Louis, MI, USA, 171261) was added to the cells (33).

Inducer (depending on the riboswitch, tetracycline, or toyocamycin) was added to cells either directly or 24 hpt (hours post-transfection). Both inducers were used from a stock solution diluted in sterile water, at a 10 mg/mL concentration for tetracycline and 1 mg/mL for toyocamycin. The final concentrations used in cell culture were 5 μM for toyocamycin, and varying, but usually 100 μM for tetracycline. Cells were generally collected at 48 hpt.

### Vector production and purification

Recombinant AAV6 vectors were produced in HEK293 cells in T175 flasks. Cells (14 × 10^6^ cells/flask) were split in DMEM with 10% FBS, 4 mM L-glutamine (Gibco 25030-024), and 50 μg/mL Pen-Strep a day before transfections. At the time of transfections, the confluency was 40–50%. Transfections were done as described above. A two-plasmid system was used with the pDP6 plasmid (Plasmid Factory, Bielefeld, Germany) and an ITR plasmid containing either the control or the riboswitch-containing sequence. These plasmids were added at a 2:1 ratio. At 24 hpt, media was changed to 0% FBS. The material was harvested at 72 hpt by adding 0.5% lysis buffer (Triton-X-100, 2 mM MgCl_2_, Merck, Rahway, New Jersey, 1.08643.1000) with SAN-HQ (Salt Active Nuclease High Quality, 100 U/mL, 70921-160 or 70920-150, ArcticZymes, Tromsø, Norway) and incubating in 37°C for 2 h, gently shaking the flasks every 30 min. Media from all flasks containing the same vector were then combined and cell debris removed by centrifuging at 1,000 *g* for 10 min.

The material was clarified with Millistak + μPod 0.0023 m^2^ (Merck MC0HC23CL3) and Sartopore 2 XLG size 4 two-layer filter (Sartorius, Göttingen, Germany 5441307G4) filters. Chromatography was performed with ÄKTA Avant 150 with POROS AAVX columns (Thermo Scientific A36651 or A36652). Elutions were neutralized with 0.5 M Tris (pH 8.8, 10% of the elution volume). The buffer was then exchanged to phosphate-buffered saline (PBS, Gibco 14190-094) by dialysis with Slide-A-Lyzer dialysis cassette (0.5–3 mL, Thermo Fisher Scientific 66382). Finally, the material was sterile filtrated through Acrodisc Syringe Supor membrane filter (0.8/0.2 μm, Pall, Port Washington, New York, NY, USA 4905).

Vectors were titered by droplet digital PCR (ddPCR) and ELISA as described previously (34). In ddPCR, viral genomes were quantified with CMV promoter primers (Supplementary Table 3). To quantify viral particles, the AAV6 Titration ELISA kit (Progen, Heidelberg, Germany PRAAV6) was used according to manufacturer instructions. Capsid protein profile was assessed by Western blot (antibodies specified below).

### Flow cytometry

Destabilized enhanced green fluorescent protein-transfected cells were collected from multi-well plates by detaching with TrypLE Select (Gibco 12563-011), pelleted by centrifuging at 500 *g* and washed with PBS. Finally, the cells were fixed with 4% paraformaldehyde (Sigma-Aldrich 158127) in PBS. The Beckman Coulter CytoFLEX S with the CytExpert software (Beckman Coulter, Brea, CA, USA) was used for the quantification of mean fluorescence intensity.

### Murine secreted alkaline phosphatase luminescence quantification

Murine secreted alkaline phosphatase was quantified by collecting cell culture media and analyzing them with the Phospha-Light System (Applied Biosystems, Waltham, MA, USA, T1017) according to the manufacturer instructions on multiwell plates. Luminescence was measured at 0.1 s per well with Varioskan Lux (Thermo Scientific) and SkanIt Software.

### Immunoblotting

VEGF-B186 was quantified from cell culture media primarily by Western blotting. Samples were heat-denatured in Laemmli sample buffer (Bio-Rad, Hercules, CA, USA, 1610747) with 2-mercaptoethanol (Sigma-Aldrich M3148). Equal volumes of samples were then run in Mini-Protean Tris-Glycine eXtended (TGX) precast gel (4–20%, Bio-Rad 456-1094) with the marker Precision Plus Protein Dual Color Standards (Bio-Rad 161-0374). The proteins were transferred into a polyvinylidene difluoride (PVDF) membrane (*Trans-*Blot Turbo 0.2 μm PVDF Transfer Pack, Bio-Rad 1704157). The membrane, after blocking with 5% non-fat dried milk in TBS-Tween20 (0.1%), was incubated with primary antibody (Anti-human VEGF-B_167/186_, R&D Systems, Minneapolis, MI, USA, AF751, RRID:AB_355571) diluted to 1:1000, overnight at 4°C, or 2 h at room temperature. Secondary antibody (Donkey Anti-Goat HRP, R&D Systems HAF109, RRID:AB_357236) was diluted to 1:2500, and the incubation was 1 h at room temperature. The proteins were revealed with Clarity Western enhanced chemiluminescence (ECL) Substrate (Bio-Rad 1705061) and imaged on ChemiDoc Touch Imaging System (Bio-Rad).

Adeno-associated virus capsid and muSEAP proteins were blotted in a similar way. For purified rAAV, 5 × 10^9^ viral genomes were loaded into each well. The primary antibody was anti-AAV VP1/VP2/VP3 mouse monoclonal, B1 (Progen 61058, RRID:AB_1540385) at 1:250 dilution, and the secondary antibody Goat Anti-Mouse IgG (H + L)-HRP Conjugate (Bio-Rad #1706516, RRID:AB_11125547), diluted 1:3000. MuSEAP antibodies were Anti-Alkaline Phosphatase antibody (Santa Cruz Biotechnology, Dallas, TX, USA, sc-398461, RRID:AB_2916293) at a 1:1000 dilution and Anti-Mouse HRP (Bio-Rad 170-6516, RRID:AB_11125547) at 1:2500.

Besides traditional Western blotting, VEGF-B186 was quantified with the Jess Simple Western assay (Biotechne, Minneapolis, MI, USA). The Separation (SM W004, Protein Simple, Biotechne) and Detection (DM-006, Protein Simple, Biotechne) Modules were prepared according to manufacturer instructions. The primary antibody (same as above) was used at a 1:50 dilution.

### BaF3-R1 cell growth and survival assay

The media from transfected 293T cells were used to verify the biological activity of the codon-optimized VEGF-B186 in BaF3-R1 cell assay. BaF3-R1 cells express chimeric VEGFR-1/EpoR receptor (35). Normally, the cells are dependent on recombinant mouse Interleukin-3 (rmIL-3) for growth and survival. However, during rmIL-3 deprivation, the activation of the chimeric receptor by VEGF family ligands can rescue the cells.

Briefly, 18,000 cells were seeded into 96-well plates in rmIL-3-free media, and dilution series of media samples were added into wells. After a 48-h incubation at 37°C, Cell Titer96 Aqueous One Solution Cell Proliferation Assay (MTS) reagent (Promega, Madison, WI, USA, G3582) was added to each well, and plates were incubated for 2 h at 37°C. Absorbances were measured at 490 and 700 nm using Varioskan Lux with SkanIt Software. The 700 nm values were subtracted from the 490 nm values.

### Statistical analysis

The data was analyzed in GraphPad Prism (version 9). The graphs present data as means with individual data points or standard deviation. The data were analyzed for normal distribution, followed by ANOVA. Further *post-hoc* tests are indicated in figure legends. Statistical significances are denoted in figures as ns (non-significant), *(*p* < 0.05), ^**^(*p* < 0.01), ^***^(*p* < 0.001), and ^****^(*p* < 0.0001).

## Data availability statement

The raw data supporting the conclusions of this article will be made available by the authors, without undue reservation.

## Author contributions

KA devised the project and the main conceptual ideas. LG designed the original plasmids. PT and PK constructed the original plasmids. RE, TN, and LG designed the experiments. RE, TN, and SP performed the experiments. HLei, IO, SL, AV, and EL produced and purified the AAVs. RE and TN analyzed the data and wrote the sections of the manuscript. RE prepared the original draft of the manuscript. RE, TN, and KA contributed to the editing of the manuscript. KA, LG, and TN designed the study and supervised the work. SY-H and HLes acquired the funding. All authors reviewed and approved the manuscript.
