# Corrigendum: Optimized riboswitch-regulated AAV vector for VEGF-B gene therapy

**DOI:** 10.3389/fmed.2023.1143748

**Published:** 2023-03-03

**Authors:** Reetta A. E. Eriksson, Tiina Nieminen, Lionel Galibert, Sanna K. Peltola, Petra Tikkanen, Piia Käyhty, Hanna M. Leinonen, Igor Oruetxebarria, Saana Lepola, Anniina J. Valkama, Eevi M. Lipponen, Hanna P. Lesch, Seppo Ylä-Herttuala, Kari J. Airenne

**Affiliations:** ^1^Kuopio Center for Gene and Cell Therapy, Kuopio, Finland; ^2^A.I. Virtanen Institute for Molecular Sciences, University of Eastern Finland, Kuopio, Finland; ^3^Gene Therapy Unit and Research Center, Kuopio University Hospital, Kuopio, Finland

**Keywords:** riboswitch, ON-switch, gene therapy, AAV (adeno-associated virus), VEGF-B, tetracycline, transgene expression regulation

In the published article, there was an error in the author list, and authors Hanna M. Leinonen, Igor Oruetxebarria, Saana Lepola, Anniina J. Valkama and Eevi M. Lipponen were erroneously excluded.

In the published article, there was an error in the Funding statement. The addition of the erroneously excluded authors “HLei,” “IO,” “SL,” “AV,” and “EL” must be added to statement. The statement previously stated:

“This study was funded by the Kuopio Center for Gene and Cell Therapy (RE, TN, LG, SP, PT, PK, HL, and KA), University of Eastern Finland Doctoral Programme for Molecular Medicine (RE), and ERC Advanced Grant (SY-H).”

The correct Funding statement appears below.

“The study was funded by Kuopio Center for Gene and Cell Therapy (RE, TN, LG, SP, PT, PK, HLei, IO, SL, AV, EL, HLes, and KA), University of Eastern Finland Doctoral Programme for Molecular Medicine (RE), and ERC Advanced Grant (SY-H).”

In the published article, there were some errors. The addition of the erroneously excluded authors has led to changes in the Conflict of Interest statement and Authors Contributions section. The Acknowledgments section has also been updated.

A correction has been made to the Conflict of Interest statement. The statement previously stated:

“RE, TN, SP, PT, PK, HL, LG, and KA were employed by Kuopio Center for Gene and Cell Therapy.

The remaining authors declare that the research was conducted in the absence of any commercial or financial relationships that could be construed as a potential conflict of interest.”

The corrected statement appears below:

“RE, TN, LG, SP, PT, PK, HLei, IO, SL, AV, EL, HLes, and KA were employed by Kuopio Center for Gene and Cell Therapy.

The remaining author declares that the research was conducted in the absence of any commercial or financial relationships that could be construed as a potential conflict of interest.”

A correction has been made to the Author Contributions section. The section previously stated:

“KA devised the project and the main conceptual ideas. LG designed the original plasmids. PT and PK constructed the original plasmids. RE, TN, and LG designed the experiments. RE, TN, and SP performed the experiments. RE and TN analyzed the data and wrote the sections of the manuscript. RE prepared the original draft of the manuscript. RE, TN, and KA contributed to the editing of the manuscript. KA, LG, and TN designed the study and supervised the work. SY-H and HL acquired the funding. All authors reviewed and approved the manuscript.”

The corrected section appears below:

“KA devised the project and the main conceptual ideas. LG designed the original plasmids. PT and PK constructed the original plasmids. RE, TN, and LG designed the experiments. RE, TN, and SP performed the experiments. HLei, IO, SL, AV, and EL produced and purified the AAVs. RE and TN analyzed the data and wrote the sections of the manuscript. RE prepared the original draft of the manuscript. RE, TN, and KA contributed to the editing of the manuscript. KA, LG, and TN designed the study and supervised the work. SY-H and HLes acquired the funding. All authors reviewed and approved the manuscript.”

A correction has been made to the Acknowledgments section. The section previously stated:

“We thank KCT upstream, downstream, and analytical teams for the help in experiments.”

The corrected section appears below:

“We thank KCT analytical teams for the help in experiments.”

The authors apologize for these errors and state that this does not change the scientific conclusions of the article in any way. The original article has been updated.

