# Supplementary material for: Optimized riboswitch-regulated AAV vector for VEGF-B gene therapy
Source: Front Med (Lausanne). 2022 Dec 13;9:1052318. doi: 10.3389/fmed.2022.1052318 (PMC9792491; doi:10.3389/fmed.2022.1052318)
Supplement: Supplementary file 1 [file Data_Sheet_1.PDF]

# *Supplementary Material*

**(A)**

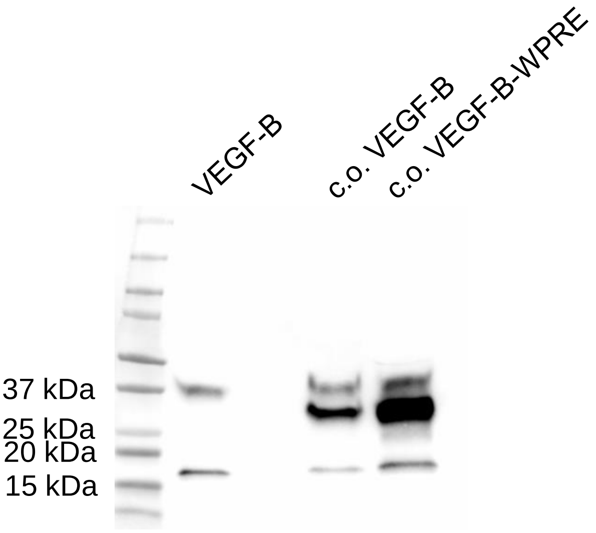


**(B)**

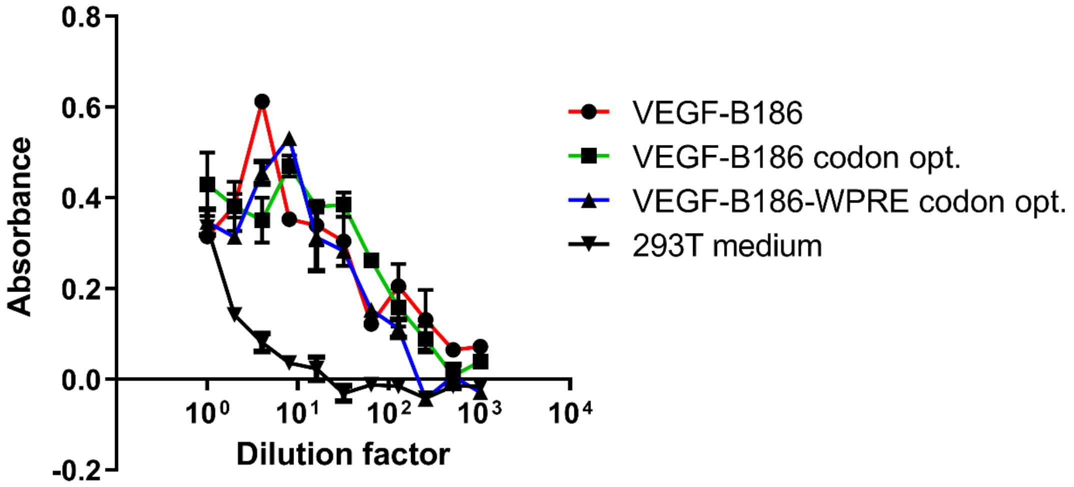


Supplementary figure 1: codon-optimization of *VEGF-B186*, and the WPRE in the codon-optimized plasmid. 293T cells were transfected with the different plasmids. **(A)** Western blot showing the increased level of VEGF-B after codon optimization, and a further increase with the added WPRE. **(B)** BaF3-R1 cell growth and survival assay showing the biological activity of VEGF-B186 protein.

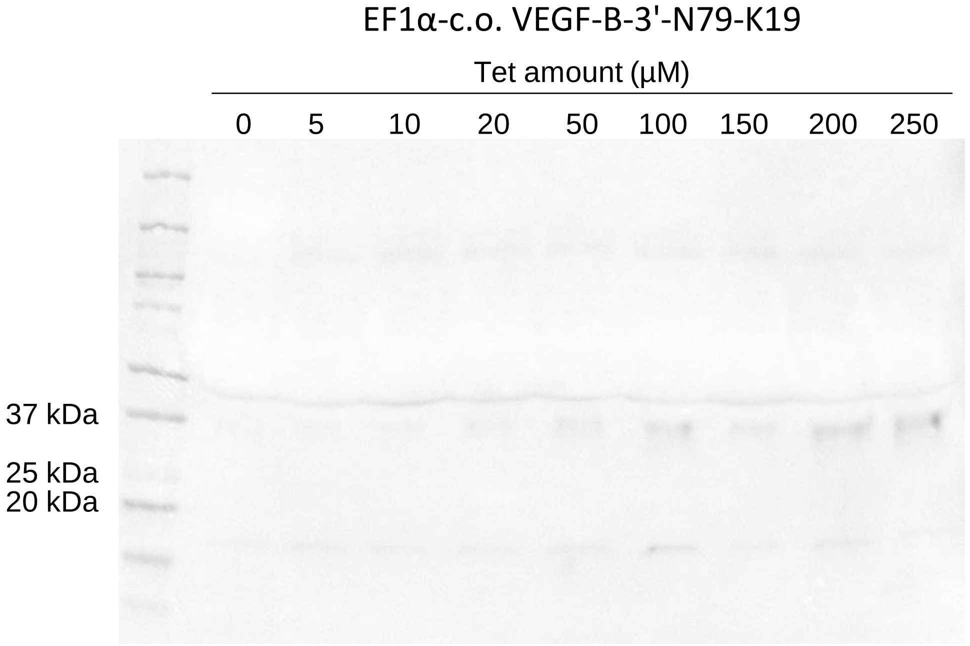


Supplementary figure 2: riboswitch-controlled EF1α promoter-driven VEGF-B186 expression was detected by Western blot, especially with high tetracycline doses, at a longer exposure time (10 minutes).

**(A)**

| Vector | ddPCR titer (vg/mL) | ELISA titer (vp/mL) | % Full |
|---|---|---|---|
| rAAV6-muSEAP | $7.59x10^{11}$ | $2.94x10^{12}$ | 26 |
| rAAV6-muSEAP-3’-N79-K19 | $1.37x10^{12}$ | $5.75x10^{12}$ | 24 |
| rAAV6-c.o.VEGF-B186 | $1.35x10^{12}$ | NA | NA |
| rAAV6-c.o.VEGF-B186-3’3’-N79-K19 | $2.81x10^{12}$ | NA | NA |

**(B)**

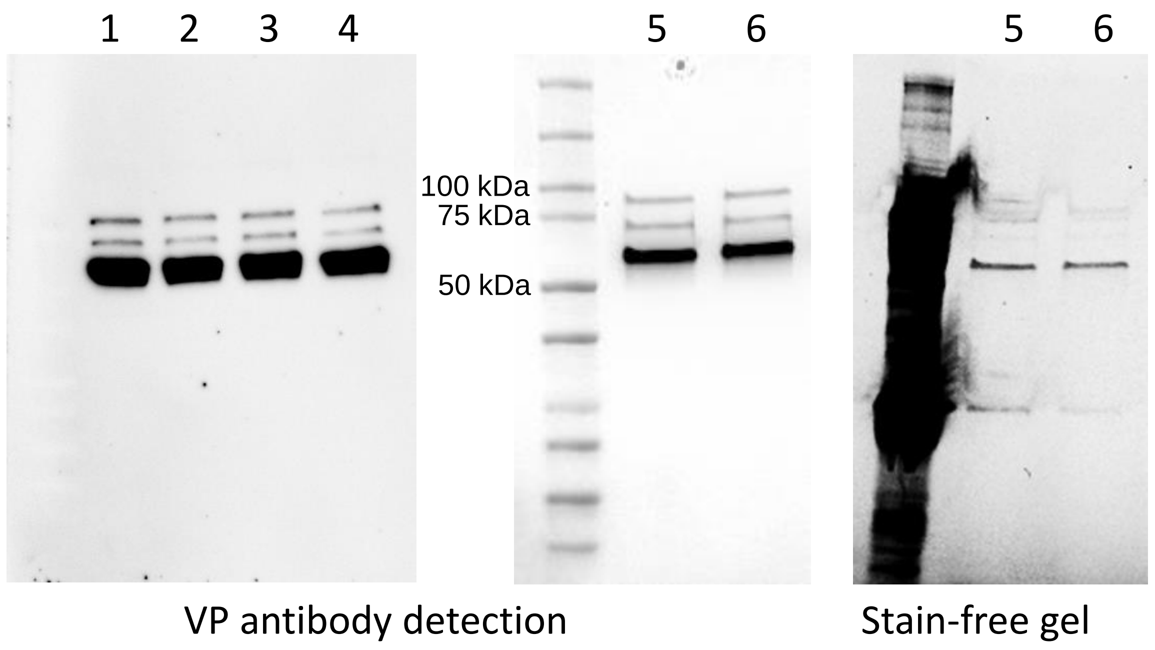


1. rAAV6-muSEAP-3’-N79-K19 pooled
2. rAAV6-muSEAP-3’-N79-K19 final
3. rAAV6-muSEAP pooled
4. rAAV6-muSEAP final
5. rAAV6-c.o.VEGF-B186
6. rAAV6-c.o.VEGF-B186-3’3’-N79-K19

Supplementary figure 3: rAAV6 vector analysis. MuSEAP and VEGF-B186 vectors were produced at different times. **(A)** Droplet digital (dd)PCR and ELISA tittering. The proportion of full particles was calculated by dividing the amount of viral genomes (vg; ddPCR) by the amount of viral particles (vp; ELISA). **(B)** Western blot. 5x109 vg was loaded into each well; staining for AAV capsid proteins. muSEAP vectors before (1 & 3) and after (2 & 4) buffer exchange and sterile filtration, and codon-optimized VEGF-B186 vectors (final products, 5 & 6).

**(A)**

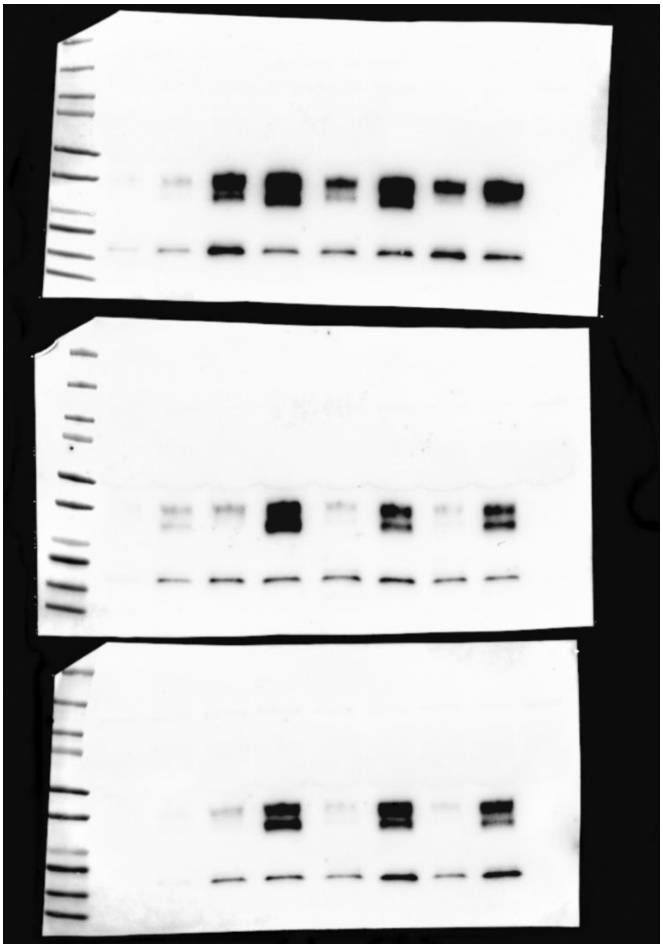

**(B)**

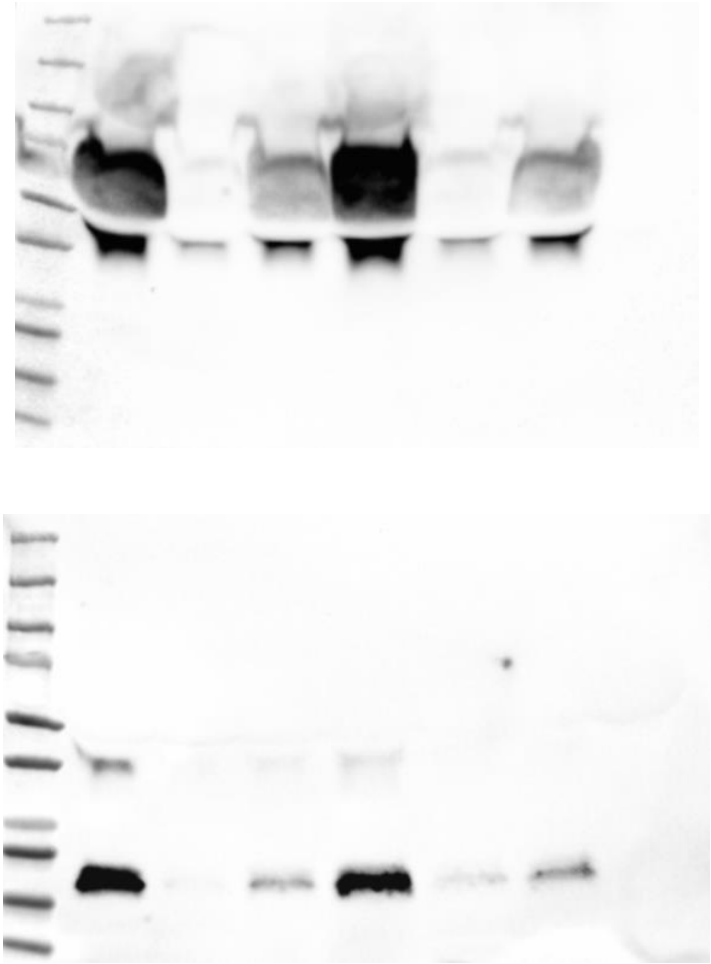

**(C)**

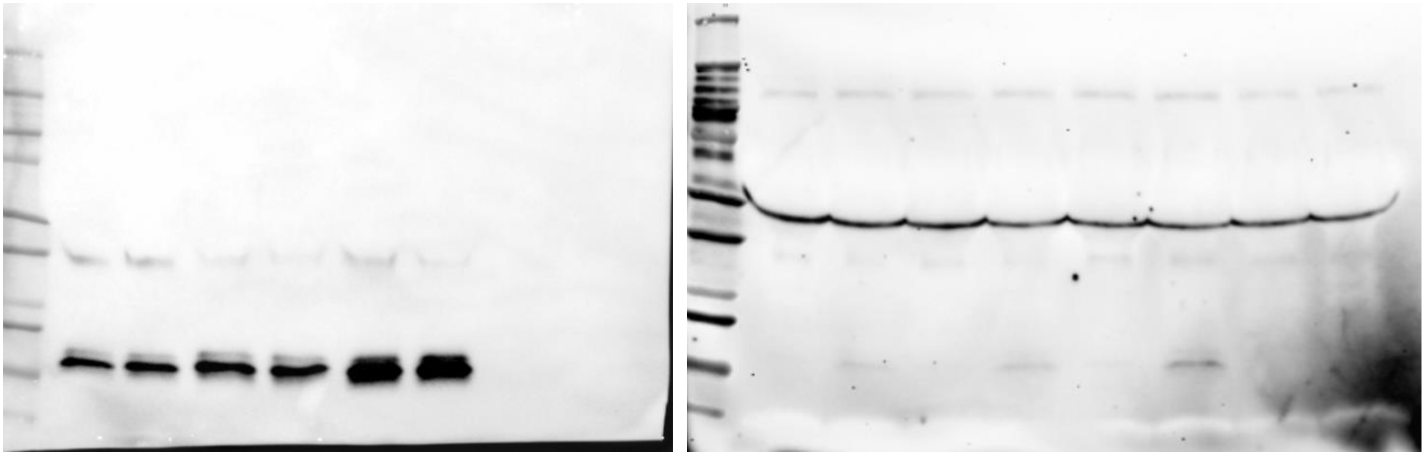

Supplementary figure 4: Full images of cropped blots. **(A)** Figure 10B **(B)** Figure 11B **(C)** Figure 11D.

# Supplementary table 1: Plasmid list

| | Plasmid number | Full name | Referred as | Size (bp) | Details |
|---|---|---|---|---|---|
| Original designs (d2EGFP with CMV-chimeric intron) | p0077 | CMV-intron-Ad-3'-SmHHrz-N79-Lio1 | 3'-N79-Lio1 | 4181 | CMV enhancer - CMV promoter - chimeric intron - d2EGFP - tet aptamer Lio1 - Schistosoma mansoni hammerhead N79 ribozyme - SV40 polyA |
| | p0078 | CMV-intron-Ad-3'-SmHHrz-N79-Tc40 | 3'-N79-TC40 | 4179 | Tet aptamer Tc40 - Schistosoma mansoni hammerhead N79 ribozyme - d2EGFP |
| | p0079 | CMV-intron-Ad-5'-SmHHrz-N79-toyo | 5'-N79-Toyo | 4142 | Toyo aptamer - Schistosoma mansoni hammerhead N79 ribozyme - d2EGFP |
| | p0080 | CMV-intron-Ad-3'-SmHHrz-N79-toyo | 3'-N79-Toyo | 4142 | Toyo aptamer - Schistosoma mansoni hammerhead N79 ribozyme - d2EGFP |
| | p0081 | CMV-intron-Ad-3'-SmHHrz-N117-toyo | 3'-N117-Toyo | 4140 | Toyo aptamer - Schistosoma mansoni hammerhead N117 ribozyme - d2EGFP |
| | p0082 | CMV-intron-Ad-5'-SmHHrz-N79-K4 | 5'-N79-K4 | 4151 | Tet aptamer K4 - Schistosoma mansoni hammerhead N79 ribozyme - d2EGFP |
| | p0083 | CMV-intron-Ad-3'-SmHHrz-N79-K4 | 3'-N79-K4 | 4151 | Tet aptamer K4 - Schistosoma mansoni hammerhead N79 ribozyme - d2EGFP |
| | p0084 | CMV-intron-Ad-5'-SmHHrz-N79-K19 | 5'-N79-K19 | 4153 | Tet aptamer K19 - Schistosoma mansoni hammerhead N79 ribozyme - d2EGFP |
| | p0085 | CMV-intron-Ad-3'-SmHHrz-N79-K19 | 3'-N79-K19 | 4153 | Tet aptamer K19 - Schistosoma mansoni hammerhead N79 ribozyme - d2EGFP |
| | p0086 | CMV-intron-Ad-5'-TRSV-HH-L2bulge18tc | 5'-TRSV-HH-L2bulge18 | 4171 | Tet aptamer L2bulge18 - Tobacco ringspot virus hammerhead ribozyme - d2EGFP |
| | p0087 | CMV-intron-Ad -3'-TRSV-HH-L2bulge18tc | 3'-TRSV-HH-L2bulge18 | 4171 | Tet aptamer L2bulge18 - Tobacco ringspot virus hammerhead ribozyme - d2EGFP |
| Promoter region modification + transgene change | p0140 | pUC57-CMV mutaORF-SV40-EGFP-3'-SmHHrz-K4tc | EGFP-3'-N79-K4 | 3896 | Tet aptamer K4 – p0083 promoter region changed to CMV enhancer - CMV promoter - Mutated - SV40 partial intron - SV40 pA in pUC57 Kana backbone |
| | p0141 | pUC57-CMV mutaORF-SV40-EGFP-3'-SmHHrz-K19tc | EGFP-3'-N79-K19 | 3898 | Tet aptamer K19 – p0085 promoter region changed to CMV enhancer - CMV promoter - Mutated - SV40 partial intron - SV40 pA in pUC57 Kana backbone |
| | p0119 | pUC57-CMV mutaORF-SV40-VEGFB186-3'-SmHHrz-N79-Lio1tc | VEGF-B-3'-N79-Lio1 | 3710 | Tet aptamer Lio1 - Schistosoma mansoni hammerhead N79 ribozyme - VEGF-B186 |
| | p0121 | pUC57-CMV mutaORF-SV40-VEGFB186-3'-SmHHrz-K4tc | VEGF-B-3'-N79-K4 | 3680 | Tet aptamer K4 - Schistosoma mansoni hammerhead N79 ribozyme - VEGF-B186 |
| | p0123 | pUC57-CMV mutaORF-SV40-VEGFB186-3'-SmHHrz-K19tc | VEGF-B-3'-N79-K19 | 3682 | Tet aptamer K19 - Schistosoma mansoni hammerhead N79 ribozyme - VEGF-B186 |
| | p0125 | pUC57-CMV mutaORF-SV40-VEGFB186-3'-TRSV-L2Bulge18tc | VEGF-B-3'-TRSV-HH-L2bulge18 | 3700 | Tet aptamer L2bulge18 - Tobacco ringspot virus hammerhead ribozyme - VEGF-B186 |
| | p0183 | pUC57-CMV mutaORF-SV40-muSEAP-3'SmHHrz-K19tc | muSEAP-3'-N79-K19 | 4576 | Tet aptamer K19 - Schistosoma mansoni hammerhead N79 ribozyme - muSEAP |
| WPRE position | p0143 | pUC57-CMV mutaORF-SV40-EGFP-3'-SmHHrz-K19tc-WPRE | EGFP-3'-N79-K19-WPRE | 4492 | Tet aptamer K19 – WPRE after riboswitch - CMV enhancer - CMV promoter - Mutated - SV40 partial intron - SV40 pA |
| | p0144 | pUC57-CMV mutaORF-SV40-EGFP-WPRE-3'-SmHHrz-K19 | EGFP-WPRE-3'-N79-K19 | 4495 | Tet aptamer K19 - WPRE before riboswitch - CMV enhancer - CMV promoter - Mutated - SV40 partial intron - SV40 pA |
| VEGFB WPRE + codon optimization | p0175 | pUC57-CMV mutaORF-SV40-VEGFB186-WPRE in pUC57-Kan | VEGF-B-WPRE | 4143 | CMV enhancer - CMV promoter - Mutated - SV40 partial intron - VEGF-B186-WPRE - SV40 pA |
| | p0145 | pUC57-CMV mutaORF-SV40-VEGF-B186-WPRE-3'-SmHHrz-K19 | VEGF-B-WPRE-3'-N79-K19 | 4273 | Tet aptamer - WPRE before riboswitch - CMV enhancer - CMV promoter - Mutated - SV40 partial intron - SV40 pA |
| | p0269 | pUC57-CMV mutaORF-SV40-codon-optimized-VEGFB186-3'-SmHHrz-K19tc | c.o. VEGF-B-3'-N79-K19 | 3681 | Tet aptamer - CMV enhancer - CMV promoter - Mutated - SV40 partial intron - codon-optimized VEGF-B186 - SV40 pA |
| | p0268 | pUC57-CMV mutaORF-SV40-codon-optimized-VEGFB186-Wpre-3'-SmHHrz-K19tc | c.o. VEGF-B-WPRE-3'-N79-K19 | 4276 | Tet aptamer - CMV enhancer - CMV promoter - Mutated - SV40 partial intron - codon-optimized VEGF-B186-Wpre - SV40 pA |

| | | | | | |
|---|---|---|---|---|---|
| CMV weak | p0363 | pUC57-CMV-weak-SV40-codon-optimized-VEGFB186-Wpre-3'-SmHHrz-K19tc | CMVweak-c.o. VEGF-B-WPRE-3'-N79-K19 | 3939 | CMV enhancer removed (almost entirely) from p0268 by digestion |
| | p0364 | pUC57-CMV-weak-SV40-codon-optimized-VEGFB186-3'-SmHHrz-K19tc | CMVweak-c.o. VEGF-B-3'-N79-K19 | 3344 | CMV enhancer removed (almost entirely) from p0269 by digestion |
| | p0366 | pUC57-CMV-weak-codon-optimized-VEGFB186-3'-SmHHrz-K19tc | CMVweak-c.o. VEGF-B-3'-N79-K19 (no SV40) | 3247 | SV40 intron removed from p0364 |
| Promoter change | p0353 | pUC57-EF1a-VEGF-B186-3'-SmHHrz-N79-K19tc codon optimized | EF1α-c.o.VEGF-B-3'N79-K19 | 4430 | Promoter changed from p0269 |
| | p0355 | pUC57-hPGK-VEGF-B186-3'-SmHHrz-N79-K19tc codon optimized | hPGK-c.o. VEGF-B-3'N79-K19 | 3773 | Promoter changed from p0269 |
| Linker length | p0312 | pUC57-CMV-SV40-VEGFB186-dual-K19tc (random 100nt) | (VEGF-B-)3'3'-N79-K19 | 3924 | Tet aptamer - dual riboswitch - random linker 100nt - CMV enhancer - CMV promoter - Mutated - SV40 partial intron - VEGF-B186 - SV40 pA |
| | p0239 | pUC57-CMV-SV40-VEGFB186-dual-K19tc (linker 100nt) | (VEGF-B-)3'3'-N79-K19 | 3924 | Dual riboswitch - linker 100nt |
| | p0313 | pUC57-CMV-SV40-VEGFB186-dual-K19tc (linker 75nt) | (VEGF-B-)3'3'-N79-K19 | 3899 | Dual riboswitch - linker 75nt |
| | p0314 | pUC57-CMV-SV40-VEGFB186-dual-K19tc (linker 50nt) | (VEGF-B-)3'3'-N79-K19 | 3874 | Dual riboswitch - linker 50nt |
| | p0315 | pUC57-CMV-SV40-VEGFB186-dual-K19tc (linker 25nt) | (VEGF-B-)3'3'-N79-K19 | 3849 | Dual riboswitch - linker 25nt |
| | p0316 | pUC57-CMV-SV40-VEGFB186-dual-K19tc (no linker) | (VEGF-B-)3'3'-N79-K19 | 3824 | Dual riboswitch - no linker added |
| | p0317 | pUC57-CMV-SV40-VEGFB186-triple-K19tc (linker 100nt) | (VEGF-B-)3'3'3'-N79-K19 | 4158 | Triple riboswitch - linker 100nt |
| Cod.opt. VEGF-B dual + triple | p0349 | pUC57-CMV mutaORF-SV40-VEGF-B186-dual-3'-SmHHrz-K19tc codon optimized (linker 100nt) | c.o. VEGF-B-3'3'-N79-K19 | 3927 | Tet aptamer - dual riboswitch - 100nt linker - CMV enhancer - CMV promoter - Mutated - SV40 partial intron - codon opt. VEGF-B186 - SV40 pA |
| | p0350 | pUC57-CMV mutaORF-SV40-VEGF-B186-triple-3'-SmHHrz-K19tc cod. opt. (linker 100 nt) | c.o. VEGF-B-3'3'3'-N79-K19 | 4161 | Tet aptamer - triple riboswitch - 100nt linker - CMV enhancer - CMV promoter - Mutated - SV40 partial intron - codon opt. VEGF-B186 - SV40 pA |
| Dual + triple riboswitch promoter comparisons | p0351 | pUC57-CMV-weak-SV40-VEGF-B186-dual-3'-SmHHrz-K19tc cod. opt. (linker 100nt) | CMVweak-c.o. VEGF-B-3'3'-N79-K19 | 3590 | CMV enhancer removed (almost entirely) from p0349 |
| | p0352 | pUC57-CMV-weak-SV40-VEGF-B186-triple-3’ SmHHrz-K19tc cod.opt. (linker 100 nt) | CMVweak-c.o. VEGF-B-3'3'3'-N79-K19 | 3824 | CMV enhancer removed (almost entirely) from p0350 |
| | p0383 | pUC57-CMV-weak-VEGF-B186-dual-3'-SmHHrz-K19tc cod.opt. (linker 100nt) | CMVweak-c.o. VEGF-B-3'3'-N79-K19 (no SV40) | 3493 | SV40 intron removed from p0351 |
| | p0384 | pUC57-CMV-weak-VEGF-B186-triple-3'-SmHHrz-K19tc cod.opt. (linker 100 nt) | CMVweak-c.o. VEGF-B-3'3'3'-N79-K19 (no SV40) | 3727 | SV40 intron removed from p0352 |
| AAV plasmids | p0303 | pAAV-VEGFB186-3'-SmHHrz-K19tc | pAAV-VEGF-B-3'-N79-K19 | 4554 | CMV enhancer CMV promoter (mutated) - SV40 partial intron - VEGFB186 tet aptamer - SV40 pA in pAAV backbone |
| | p0304 | pAAV-VEGFB186 | pAAV-VEGF-B | 4420 | CMV enhancer CMV promoter (mutated) - SV40 partial intron VEGFB186 - SV40 pA in pAAV backbone |
| | p0305 | pAAV-muSEAP-3'-SmHHrz-K19tc | pAAV-muSEAP-3'-N79-K19 | 5448 | CMV enhancer CMV promoter (mutated) - SV40 partial intron - muSEAP tet aptamer - SV40 pA in pAAV backbone |
| | p0306 | pAAV-muSEAP | pAAV-muSEAP | 5301 | CMV enhancer CMV promoter (mutated) - SV40 partial intron - muSEAP - SV40 pA in pAAV backbone |
| | p0403 | pAAV-VEGF-B186-dual-3'-SmHHrz-K19tc codon optimized (linker 100nt) | pAAV-c.o. VEGF-B-3'3'-N79-K19 | 4743 | CMV enhancer CMV promoter (mutated) - SV40 partial intron - codon optimized VEGF-B186 - 2x 3' K19 - 100 nt linker - SV40 pA in pAAV backbone |

Supplementary table 2: Linker sequences.

| | |
|---|---|
| 25 nt | ggacgcacgctatgagggccttatc |
| 50 nt | ggacgcacgctatgagggccttatccttattacctataaattgttccacc |
| 75 nt | ggacgcacgctatgagggccttatccttattacctataaattgttccaccgaacattcttcaaaatggtaaaacc |
| 100 nt | ggacgcacgctatgagggccttatccttattacctataaattgttccaccgaacattcttcaaaatggtaaaacctattgacttgaccgtcaacagactg |
| 100 nt random | agagggtaatcagccgtgttcacctacacaacgctaacgggcgattctataagattccgcattgcgtctacttataagatgtctcaacggtatccgcaac |

Supplementary table 3: ddPCR primer and probe sequences.

| | |
|---|---|
| CMV forward | 5'-CAT GAC CTT ATG GGA CTT TCC T-3' |
| CMV reverse | 5'-CTA TCC ACG CCC ATT GAT GTA-3' |
| CMV probe | 5'-/56-FAM/TCG CTA TTA/ZEN/CCA TGG TGATGC GGT/3IABkFQ/-3' |
